# HALT-IT - tranexamic acid for the treatment of gastrointestinal bleeding: study protocol for a randomised controlled trial

**DOI:** 10.1186/1745-6215-15-450

**Published:** 2014-11-19

**Authors:** Ian Roberts, Timothy Coats, Phil Edwards, Ian Gilmore, Vipul Jairath, Katharine Ker, Daniela Manno, Haleema Shakur, Simon Stanworth, Andrew Veitch

**Affiliations:** Clinical Trials Unit, London School of Hygiene & Tropical Medicine, Keppel Street, London, WC1E 7HT UK; Department of Cardiovascular Sciences, University of Leicester, Level G, Jarvis Building RMO, Infirmary Square, Leicester, LE1 5WW UK; Royal Liverpool University Hospital, Prescot Street, Liverpool, L7 8XP UK; Translational Gastroenterology Unit, John Radcliffe Hospital, Oxford, OX3 9DU UK; NHS Blood & Transplant, John Radcliffe Hospital, Oxford, OX3 9DU UK; Endoscopy & Bowel Cancer Screening Department, New Cross Hospital, Wolverhampton, WV10 0QP UK

**Keywords:** Gastrointestinal bleeding, Tranexamic acid, Clinical trials

## Abstract

**Background:**

Gastrointestinal bleeding is a common emergency that causes substantial mortality worldwide. Acute upper and lower gastrointestinal bleeding accounts for about 75,000 hospital admissions each year in the UK and causes the death of about 10% of these patients.

Tranexamic acid has been shown to reduce the need for blood transfusion in surgical patients and to reduce mortality in bleeding trauma patients, with no apparent increase in thromboembolic events.

A systematic review of clinical trials of upper gastrointestinal bleeding shows a reduction in the risk of death with tranexamic acid but the quality of the trials was poor and the estimates are imprecise. The trials were also too small to assess the effect of tranexamic acid on thromboembolic events.

**Methods:**

HALT-IT is a pragmatic, randomised, double-blind, placebo-controlled trial which will determine the effect of tranexamic acid on mortality, morbidity (re-bleeding, non-fatal vascular events), blood transfusion, surgical intervention, and health status in patients with acute gastrointestinal bleeding. Eight thousand adult patients who fulfil the eligibility criteria will be randomised to receive tranexamic acid or placebo.

Adults with significant acute upper or lower gastrointestinal bleeding can be included if the responsible doctor is substantially uncertain as to whether or not to use tranexamic acid in that particular patient.

Trial treatment consists of a loading dose of tranexamic acid (1 g by intravenous injection) or placebo (sodium chloride 0.9%) given as soon as possible after randomisation, followed by an intravenous infusion of 3 g tranexamic acid or placebo (sodium chloride 0.9%) over 24 hours.

The main analyses will compare those allocated tranexamic acid with those allocated placebo, on an intention-to-treat basis. Results will be presented as effect estimates with a measure of precision (95% confidence intervals). Subgroup analyses for the primary outcome will be based on time to treatment, source of bleeding (upper versus lower), suspected variceal bleeding and severity of bleeding. A study with 8,000 patients will have over 90% power to detect a 25% reduction in mortality from 10% to 7.5%.

**Trial registration:**

Current Controlled Trials ISRCTN11225767 (registration date: 3 July 2012); Clinicaltrials.gov NCT01658124 (registration date: 26 July 2012).

**Electronic supplementary material:**

The online version of this article (doi:10.1186/1745-6215-15-450) contains supplementary material, which is available to authorized users.

## Background

Acute gastrointestinal (GI) bleeding is a common emergency and an important cause of mortality and morbidity worldwide. Acute upper GI bleeding accounts for about 60,000 hospital admissions each year in the UK and has a case fatality of about 10% [1, 2]. Lower GI bleeding accounts for about 15,000 admissions each year with a case fatality of about 15% [3]. GI bleeding is also common in low- and middle-income countries, where patients are usually young and poor.

Common causes of acute upper GI bleeding in high-income countries are ulcers (40%) and oesophageal varices (11%) [2]. In low- and middle-income countries variceal bleeding is particularly common (45%), with peptic ulcers accounting for about 30% of cases. In sub-Saharan Africa, schistosomiasis is an important cause of portal hypertension, responsible for about 130,000 deaths from haematemesis each year [4]. Despite advances in the management of upper GI bleeding in the past two decades, mortality remains high. In a recent nationwide UK study, the case fatality for new presentations to hospital was 7%, rising to over 26% in patients already hospitalised for another condition [2, 5].

A strong predictor of mortality in patients with upper GI bleeding is re-bleeding, which occurs in about 10% of non-variceal [5, 6] and 25% of variceal bleeding [7, 8]. A study in patients with bleeding peptic ulcers [9] found that more than half of the re-bleeds occurred in the 24 hours after initial treatment. Re-bleeding rates have not changed significantly over the past 15 years [2, 10, 11] and ongoing research should focus on improving this outcome [10].

Leading causes of lower GI bleeding are diverticular disease, colitis and cancer [12]. Mortality from lower GI bleeding is less than 5% but increases to about 20% in patients who bleed during admission to hospital for other reasons [13]. Most cases occur in the elderly and many are associated with the use of non-steroidal anti-inflammatory drugs [3, 14].

Tranexamic acid (TXA) is commonly given to patients either before or during surgery to reduce bleeding and the need for blood transfusion. A systematic review of randomised controlled trials of TXA in surgical patients [15] shows that it reduces the probability of receiving a blood transfusion by about a third (risk ratio (RR) = 0.62, 95% CI 0.58 to 0.65), with no evidence of an increase in risk of thromboembolic events.

TXA has been shown to reduce mortality in bleeding trauma patients. The CRASH-2 trial, which enrolled 20,211 patients from hospitals in 40 countries, shows that the administration of TXA within 8 hours of injury reduces deaths due to bleeding (RR = 0.85, 95% CI 0.76 to 0.96), and all-cause mortality (RR = 0.91, 95% CI 0.85 to 0.97) compared to placebo, with no apparent increase in thromboembolic events [16]. Among patients treated soon after injury, the reduction in mortality with TXA is even greater [17]. Cost-effectiveness analysis reveals that the administration of TXA to bleeding trauma patients is highly cost-effective [18]. As a consequence of the CRASH-2 trial results, TXA has been incorporated into trauma treatment protocols worldwide and is included on the World Health Organization List of Essential Medicines [19].

The knowledge that TXA reduces blood loss in surgery and reduces mortality in traumatic bleeding raises the possibility that it might also be effective for GI bleeding.

A recent update of a systematic review identified nine randomised comparisons from eight clinical trials of the use of TXA in upper GI bleeding, and none in lower GI bleeding [20]. The pooled result shows a statistically significant reduction in the risk of death in patients receiving TXA (RR = 0.66, 95% CI 0.47 to 0.93). However, the quality of the trials was poor and the estimate is imprecise. Only one trial had adequate allocation concealment. In several trials, patients were excluded after randomisation and information on their outcomes was not reported, raising the possibility of selection bias. All but two trials were conducted before the widespread use of therapeutic endoscopy and proton pump inhibitors. Moreover, the sample size of trials, even when combined in the meta-analysis, was inadequate [20]. Thus, although the meta-analysis result is statistically significant (*P* <0.05), this could easily be a false positive.

Only three trials reported data on adverse events. These studies were already included in a previous Cochrane review [21]. The risk of thromboembolic events is about 1% overall and appeared to be higher in TXA-treated patients (RR = 1.86, 95% CI 0.66 to 5.24). However, the trials are too small to assess the effect of TXA on thromboembolic events.

For these reasons, the effectiveness and safety of TXA for GI bleeding is uncertain and it is not routinely used for treatment. In a UK audit in 2007, less than 1% of patients with upper GI bleeding were given TXA [5]. TXA is not referred to in two recent international consensus documents on the management of GI bleeding [22, 23], nor in the 2012 UK National Institute for Health and Clinical Excellence guidelines for acute upper GI bleeding [24].

### Need for a trial

The HALT-IT trial will help to determine whether or not TXA should be used in the treatment of GI bleeding. If TXA reduces mortality in patients with GI bleeding, this would be of considerable significance worldwide. TXA might also reduce the need for transfusion. Blood is a scarce resource with a risk of transfusion transmitted infections.

The results will be disseminated in peer-reviewed medical journals, conference presentations, and in an updated systematic review of treatments for GI bleeding. There is evidence that hospitals participating in multi-centre trials are more likely to implement the trial results [25]. For this reason, an international multi-centre trial like the HALT-IT trial could have a substantial impact on clinical practice. The large network of collaborating sites will help to ensure that the results are disseminated worldwide.

### Tranexamic acid and its effect on bleeding

In normal haemostasis, coagulation occurs rapidly at the site of a damaged blood vessel forming a stable fibrin blood clot. However, fibrinolytic enzymes in the blood can impair clot stability and worsen bleeding [26]. TXA inhibits fibrinolytic enzymes and can thus enhance the ability to form stable blood clots.

Fibrinolysis may play an important role in GI bleeding due to the premature breakdown of fibrin blood clots at the bleeding site [27, 28]. Studies have shown that many patients with acute upper GI bleeding have elevated levels of fibrin degradation products (a surrogate marker for fibrinolysis) and that this is associated with worse outcomes [27, 28]. Fibrinolysis may also increase the risk of re-bleeding.

TXA reduces blood loss and the need for transfusion when administered before and during surgery and increases survival in traumatic bleeding, especially when given soon after injury. Early administration in patients with acute GI bleeding could possibly reduce the duration and amount of bleeding at presentation and the risk of re-bleeding by stabilising blood clots at the bleeding site. This could reduce mortality and the need for blood transfusion.

### Potential side effects of tranexamic acid

The systematic review of TXA in surgery provides no evidence for any increase in the risk of thromboembolic events in patients given TXA [15]. There was no increase in the risk of thromboembolic events in patients treated with TXA in the CRASH-2 trial [16, 17]. Indeed, there were fewer vascular occlusive deaths with TXA (RR = 0.69, 95% CI 0.44 to 1.07) and there was a statistically significant reduction in fatal and non-fatal myocardial infarction (RR = 0.64, 95% CI 0.42 to 0.97). We do not know whether TXA increases or decreases the risk of thromboembolic events in patients with GI bleeding. The trials to date are too small to assess the effect of TXA on these outcomes [21].

TXA is not a new drug. Adverse events are uncommon and usually manifest as nausea or diarrhoea, or occasionally as orthostatic reactions [29]. These symptoms are commonly associated with GI bleeding. There is some evidence from observational studies that high-dose TXA is associated with an increased risk of seizures in patients undergoing cardiac surgery [30–33]. The doses of TXA used in these studies (total doses from 7.5 g up to 20 g) are much higher than that proposed in the HALT-IT trial (4 g). An association between TXA and seizures has not been confirmed in randomised trials.

### Objective

The HALT-IT trial will provide reliable evidence as to whether early administration of TXA reduces mortality and other clinical outcomes in patients with significant acute GI bleeding.

## Methods and design

### Overview

**Figure 1:**
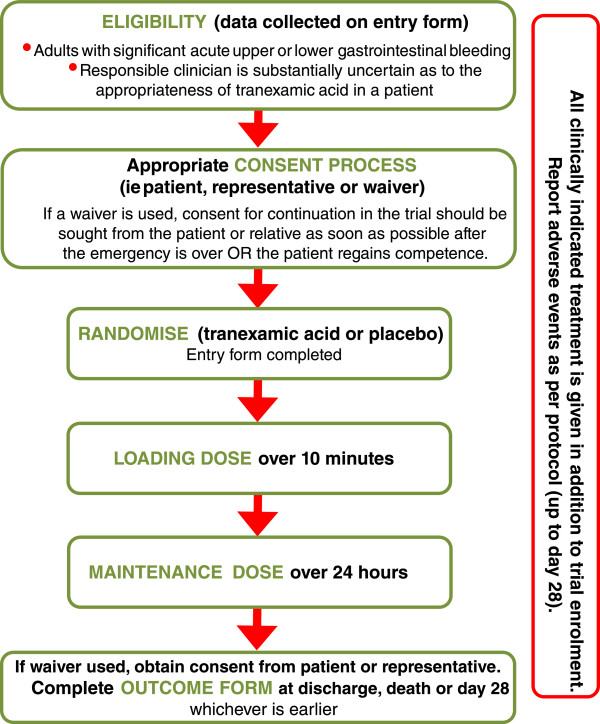
**Trial overview.**

HALT-IT trial is a large, pragmatic, randomised, double-blind, placebo-controlled trial to quantify the effects of the early administration of TXA on death, blood transfusion and other relevant outcomes. About 8,000 adults, who have significant upper or lower GI bleeding and who fulfil the eligibility criteria, will be randomised to receive either TXA or placebo. The eligibility criteria are based on the uncertainty principle. An overview of the trial is provided in Figure 1.

#### Pragmatic design and the uncertainty principle

The pragmatic design will allow us to find out how effective the treatment actually is in routine practice. The eligibility criteria are based on the uncertainty principle, which is a well established approach to trial eligibility [34]. A patient can be enrolled if, and only if, the responsible clinician is substantially uncertain as to which trial treatment would be most appropriate for that particular patient. A patient should not be enrolled if the responsible clinician or the patient (or his/her representative) are for any medical or non-medical reasons reasonably certain that one of the two allocated treatments (TXA or placebo) would not be appropriate for this particular individual (in comparison with either no treatment or some other treatment that could be offered). Clinicians, patients and their representatives will be provided with information about the trial treatment to assist them in their judgement.

#### Randomisation

Patients eligible should be randomised as soon as possible, and the study treatment started immediately. The Entry form (Additional file 1: Form 1) is used to assess eligibility and collect baseline information. The next consecutively numbered treatment pack, taken from a box of eight packs, should be chosen. Once a patient has been randomised, the outcome in hospital needs to be collected even if the trial treatment is interrupted or is not actually given.

#### Follow-up

No extra tests are required but a short Outcome form (Additional file 2: Form 2) must be completed from the medical records 28 days after randomisation or on discharge from the randomising hospital or on death (whichever occurs first). Any adverse events which become known to the investigator will be reported up to 28 days after randomisation. In England and Wales, the status (death, hospital readmission) of patients at 12 months will also be ascertained.

### Settings

The pragmatic nature of this trial will allow for the recruitment of patients from a wide variety of healthcare facilities. Participating hospitals will be selected worldwide. There is no limit to the maximum number of patients to be recruited at each site.

### Number of patients needed

Two factors determine the number of patients needed in a trial: the estimated event rate and size of the treatment effect.

#### Estimated event rate

Previous studies on GI bleeding suggest an overall mortality of 8 to 16% [35]. About 10% of patients with GI bleeding die in hospital [2, 5]. Based on these estimates, a baseline event rate of 10% mortality might reasonably be expected.

#### Sample size and size of treatment effect that should be detectable

Assuming a control group mortality rate of 10%, a study with 8,000 patients would have over 90% power (two sided alpha = 5%) to detect a clinically important 25% reduction from 10% to 7.5% in mortality. Experience from the CRASH-1 and CRASH-2 clinical trials suggests that the anticipated rate of loss to follow-up (less than 1%) would not impact importantly on study power.

### Recruitment of collaborating investigators

The trial is recruiting hospitals worldwide and will continue to add sites to ensure the sample size is achieved. Suitable collaborating sites and investigators are assessed on the number of potentially eligible patients and their ability to conduct the trial. In advance of the trial starting at a site, the Principal Investigator must agree to follow Good Clinical Practice Guidelines and all relevant regulations in their country. All relevant regulatory and ethics approvals must be in place. A hospital is not considered suitable for participating in the HALT-IT trial if TXA is in routine use for the treatment of GI bleeding, including where TXA is either mandated or recommended for GI bleeding in a massive haemorrhage treatment protocol.

### Eligibility

#### Inclusion criteria

All adults with significant acute upper or lower GI bleeding are eligible if the responsible clinician is substantially uncertain as to whether or not to use TXA and when consent has been obtained according to approved procedures.

The diagnosis of significant bleeding is clinical but *may* include patients with hypotension, tachycardia, or those likely to need transfusion, urgent endoscopy or surgery. The fundamental eligibility criterion is the responsible clinician’s ‘uncertainty’ as to whether or not to use TXA in a particular patient with GI bleeding.

#### Exclusion criteria

Patients for whom the responsible clinician considers there is a clear indication or a clear contraindication to TXA should not be randomised.

### Consent and ethical considerations

Significant acute GI bleeding is an emergency and the priority is to provide appropriate emergency care. Eligible patients have a life-threatening condition. Their physical, mental and emotional state may be affected by their blood loss. Because randomisation and administration of the trial treatment should be done as early as possible once significant GI bleeding is suspected, the consent process in this situation requires careful consideration bearing in mind applicable regulatory requirements, adherence to International Conference on Harmonisation of Good Clinical Practice (ICH-GCP), and the requirements in the Declaration of Helsinki.

#### Prior information giving

Bearing in mind the clinical situation and their level of distress, the patient and, if present, the patient’s relative will be provided with brief information about the trial. The responsible doctor will explain to the patient and relative that the patient will receive the usual emergency treatments for GI bleeding but that in addition to these, if they agree, the patient will be enrolled in a research study that aims to improve the treatment of patients with this condition. It will be explained that the study is being conducted to see whether using a drug called TXA will help patients with GI bleeding. The patient/relative will be informed that the patient will be given an infusion into a vein over 24 hours of either TXA or a dummy medicine (a liquid which does not contain TXA). The doctor will explain that TXA has been shown to improve outcome in patients with other types of severe bleeding and that whilst we hope that it will also improve recovery after GI bleeding, at present we cannot be sure about this. A brief information leaflet will be provided (Additional file 3: Form 3). If the patient or relative objects to the inclusion of the patient in the trial, his/her views will be respected.

The process by which information will be given and consent obtained will depend on the need for urgent clinical intervention and the patient’s physical, mental and emotional state. Factors which may impair the patient’s decision-making process including altered level of consciousness due to a degree of blood loss or co-morbidities (for example, liver failure) will be taken into consideration. Also, the availability of a personal representative and his/her ability to make a decision on the patient’s behalf will have to be taken into consideration. The approach that allows the patient to have the most input into the decision-making process without endangering his/her life will be utilised.

If the patient is fully competent, he/she will be approached at the time of diagnosis. The Information Sheet (Additional file 4: Form 4) will be provided, the study will be discussed with the patient and a written consent obtained (Additional file 5: Form 5). If the patient is unable to read or write, then the information sheet may be read to him/her and s/he may then mark the consent form with either a cross or thumbprint. In this event, a witness not associated with the trial, must provide a full signature confirming the mark.

If the patient’s mental capacity is impaired and either a personal or professional representative is available, then information should be given to the patient taking his/her level of mental impairment into consideration. Refusal by the patient should be respected and s/he should not be enrolled.

If a personal representative (PeR) who is knowledgeable about the patient’s values and beliefs is available, the Information Sheet will be provided (Additional file 4: Form 4). Opportunity for questions will be given and written consent obtained (Additional file 5: Form 5). If the PeR is unable to read or write, then the information sheet may be read to him/her and a mark with either a cross or thumbprint made on the consent form. In this event, a witness not associated with the trial, must provide a full signature confirming the mark.

If a PeR is not available and the patient is unable to provide valid informed consent, then an independent doctor or other site staff allowed to fulfil this role (ideally the primary carer if not part of the trial team) may be asked to consent as a professional representative (PrR). Informed consent given by a representative shall represent the patient’s presumed will.

If the patient’s mental capacity is impaired and neither a PeR or PrR is available then information should be given to the patient taking his/her level of mental impairment into consideration. Refusal by the patient should be respected and s/he should not be enrolled.

The investigator and one independent person (doctor or nurse) who is not participating in this trial may enrol the patient into the trial by certifying in writing in the patient’s medical records that: the patient has significant gastrointestinal bleeding; the patient is unable to give consent as a result of his/her medical condition; it is not feasible to contact the patient’s PeR/PrR to obtain consent; and neither the patient nor the patient’s PeR/PrR nor any member of the family has informed the investigator of any objections to the patient being enrolled as a participant in this trial.

For patients enrolled under such an emergency consent procedure, the patient or his/her PeR or PrR will be informed about the trial as soon as it is possible. Consent will be obtained for the continuation of any trial procedure. If a patient or representative declines to give consent for continuation at this stage, his/her wishes will be respected.

**Figure 2:**
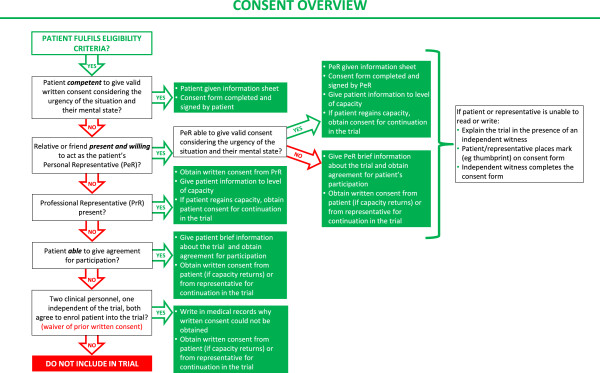
**Consent procedure diagram.**

A summary overview of the consent procedure is provided in Figure 2. The requirements of the relevant ethics committee will be adhered to at all times. Ethics approval has already been obtained from several institutions (see list of ethical bodies that approved the study in Additional file 6).

### Randomisation

Randomisation codes have been generated and secured by an independent statistician from Sealed Envelope Ltd (London, UK). The codes have been made available to a Good Manufacturing Practice (GMP) certified clinical trial supply company, which has prepared the treatment packs in accordance with the randomisation list. Eligibility will be determined from routinely collected clinical information and recorded on the trial Entry form. No trial-specific tests are required. Patients eligible for inclusion should be randomised as soon as possible to TXA or placebo by taking the next lowest consecutively numbered pack from a box of eight treatment packs. At the point when all the treatment ampoules are confirmed as being intact, the patient is considered randomised onto the trial and the trial treatment must be started immediately.

Once a patient has been randomised, the Entry form data will be sent to the Trial Coordinating Centre (TCC) as soon as possible and the outcome of the patient should be obtained even if the trial treatment is interrupted or is not actually given.

### Treatment

TXA (4 g) will be compared with matching placebo (sodium chloride 0.9%).

### Dose selection

In randomised trials in cardiac surgery, TXA dose regimens vary widely. Loading doses range from 2.5 mg/kg to 100 mg/kg and maintenance doses from 0.25 mg/kg/hour to 4 mg/kg/hour given over periods of 1 to 12 hours [36]. A loading dose of 10 mg/kg TXA followed by an infusion of 1 mg/kg/hour has been shown to produce plasma concentrations sufficient to inhibit fibrinolysis *in vitro* [37].

In the emergency situation, the administration of a fixed dose is more practicable since weighing patients is difficult. In the CRASH-2 trial, a fixed loading dose of 1 g TXA followed by a 1 g maintenance dose over eight hours was found to reduce mortality in bleeding trauma patients with no evidence of significant adverse effects [16, 17].

In the HALT-IT trial, a fixed loading dosage of 1 g TXA followed by 3 g infused over 24 hours has been selected. This dosage is within the range that has been shown to inhibit fibrinolysis [37]. It would be efficacious for larger patients (>100 kg) but also safe in smaller patients (<50 kg), as the estimated dose/kg that the patients in the latter group would receive has been applied in other trials without significant adverse effects [36, 37]. The loading dose (1 g) is the same as used in the CRASH-2 trial [16]. A maintenance dose is provided, but over a longer duration (24 hours) than used in the CRASH-2 trial, to cover the period with the greatest risk of re-bleeding.

### Drug manufacture, blinding and supply of trial treatment

TXA (Cyklokapron® Injection) is purchased on the open market in the UK. TXA is manufactured by Pfizer Ltd under Marketing Authorisation Number PL 00057/0952. The Marketing Authorisation guarantees that the product has been manufactured and released in accordance with the UK’s GMP regulations.

Placebo (sodium chloride 0.9%) is manufactured to match the TXA by a GMP certified manufacturer.

Ampoules and packaging are identical in appearance. The blinding process and first-stage qualified person release is performed by the designated clinical trial supply company. The blinding process involves complete removal of the original manufacturer’s label and replacement with the clinical trial label bearing the randomisation number which is used as the pack identification. Other pack label text is identical for both TXA and placebo treatments and is in compliance with requirements for investigational medicinal products.

The designated clinical trial supply company is also be responsible for maintaining the Product Specification File until final database lock and unblinding of the trial data. Quality control checks to assure the blinding process are performed on a random sample of final qualified person released drug packs. High performance liquid chromatography separation of known TXA is assessed against blinded samples to confirm which ampoule contains the placebo and active treatments. The tested samples are unblinded to assure accuracy of blinding.

The TCC is responsible for assuring all relevant approvals are available at the TCC before release of the trial treatment to a site. A separate Manual of Operating Procedures details the drug accountability system. The Investigator’s Brochure details labelling of the trial treatment and other processes for assuring adherence to GMP.

### Administration of trial treatment

Each treatment pack contains 8 × 500 mg ampoules of tranexamic acid or placebo, and 2 × sterile 10 ml syringes and 21 F needles.

#### Loading dose

Two ampoules = 1 g, added to 100 ml sodium chloride 0.9% and infused over 10 minutes.

#### Maintenance dose

Six ampoules = 3 g, added to 1,000 ml of any isotonic intravenous solution and infused at 125 mg/hour (42 ml/hour) for about 24 hours.

The trial treatment injections should not be mixed with blood for transfusion or infusion solutions containing penicillin or mannitol.

The loading dose of the trial treatment must be administered by intravenous infusion immediately after randomisation. The maintenance dose (by intravenous infusion) should commence as soon as the loading dose is completed.

### Other treatments for gastrointestinal bleeding

As the trial will be conducted worldwide, each participating site should follow its own clinical practice for the treatment of GI bleeding. Information on other treatments given will be collected on the Outcome form. TXA or placebo would be an additional treatment to the routine management of GI bleeding.

### Adverse events

TXA is not a new drug and has a documented safety profile. Although the Summary of Product Characteristics suggests that rare cases of thromboembolic events and seizures might be associated with TXA administration, there is no evidence that the TXA treatment regimen used in this trial is associated with an increased risk of thromboembolic events or seizures.

Data on thromboembolic events (such as deep vein thrombosis, pulmonary embolism, myocardial infarction, stroke), seizures, other significant cardiac event, respiratory, liver and renal failure are collected as secondary outcomes up to day 28 after randomisation and will be presented to the independent Data Monitoring Committee (DMC) for unblinded review.

#### Adverse event

An adverse event is any untoward medical occurrence affecting a trial participant during the course of a clinical trial.

#### Serious adverse event

A serious adverse event/experience (SAE) is any untoward medical occurrence that, at any dose: results in death; is life-threatening; requires inpatient hospitalisation or prolongation of existing hospitalisation; results in persistent or significant disability/incapacity; or is a congenital anomaly/birth defect.

#### Adverse reaction

An adverse reaction is an adverse event when there is at least a possibility that it is causally linked to a trial drug or intervention.

#### Serious adverse reaction

A serious adverse reaction is a SAE that is thought to be causally linked to a trial drug or intervention.

#### Suspected unexpected serious adverse reaction

A suspected unexpected serious adverse reaction is an unexpected occurrence of a serious adverse reaction; there need only be an index of suspicion that the event is a previously unreported reaction to a trial drug or a previously reported but exaggerated or unexpectedly frequent adverse drug reaction.

#### Reporting of adverse events for this trial

Death and life-threatening complications are pre-specified outcomes to be reported in this trial and also to the independent DMC. This clinical trial is being conducted in a critical emergency condition, using a drug in common use. It is important to consider the natural history of the critical medical event affecting each patient enrolled, the expected complications of this event and the relevance of the complications to TXA.

Adverse events to be reported using an adverse event reporting form are limited to those not already listed as primary or secondary outcomes, yet which might reasonably occur as a consequence of the trial drug. Events that are part of the natural history of GI bleeding or expected complications of this condition should not be reported as adverse events.

In addition, if a patient is discharged from the randomising hospital before day 28 and is readmitted to hospital, requires medical care for any reason, or is known to have died, an adverse event reporting form should be completed irrespective of the cause.

If a SAE occurs, reporting advice can be obtained by calling the TCC Emergency Helpline and a written report must be submitted within 24 hours. The TCC will coordinate the reporting of all SAEs to all relevant Regulatory Agencies, Ethics Committees and local investigators as per local legal requirements.

### Unblinding

In general there should be no need to unblind the allocated treatment. If some contraindication to TXA develops after randomisation (for example, the patient becomes anuric and the clinical team is concerned about acute renal failure and risk of TXA accumulation), the trial treatment should simply be stopped and all usual standard care given. Unblinding should be done only in those rare cases when the clinician believes that clinical management depends importantly upon knowledge of whether the patient received TXA or placebo. In those few cases when urgent unblinding is considered necessary, a 24-hour telephone service will be available and details provided in the Investigator’s Study File and wall posters. The caller will be informed whether the patient received TXA or placebo. An unblinding report form should be completed by the investigator.

### Measures of outcome

After a patient has been randomised, outcome in hospital will be collected even if the trial treatment is interrupted or is not actually given. No extra tests are required but a single page Outcome form (Additional file 2: Form 2) will be completed 28 days after randomisation, at discharge from the randomising hospital, or at death (whichever occurs first).

In England and Wales, mortality and hospital readmission data will also be obtained 12 months after randomisation. For deaths, the National Health Service (NHS) Information Centre service will be used to identify the date and cause of death in England. For readmissions, the NHS Information Centre Trusted Data Linkage service will be used to provide a dataset of patients linked to the Hospital Episodes Statistics dataset, including diagnoses, procedures and reason for admission. For Wales, these data will be obtained through the Secure Anonymised Information Linkage Databank.

### Primary outcome

The primary outcome is death in hospital within 28 days after randomisation (cause-specific mortality will also be recorded).

### Secondary outcomes

Secondary outcomes are: 1) re-bleeding; 2) need for surgery or radiological intervention; 3) blood product transfusion; 4) thromboembolic events (deep vein thrombosis, pulmonary embolism, stroke, myocardial infarction); 5) other complications (including other significant cardiac event, sepsis, pneumonia, respiratory failure, renal failure, liver failure, seizures); 6) functional status will be measured by the Katz Index of Independence in Activities of Daily Living [38] at discharge from the randomising hospital or in-hospital at 28 days after randomisation. The Index assesses adequacy of performance in six functions of bathing, dressing, toileting, transferring, continence and feeding. Patients are scored ‘yes’ or ‘no’ for independence in each of the functions (score of 6 = full function, 4 = moderate impairment, and ≤2 = severe functional impairment); 7) days spent in intensive care unit or high dependency unit; and 8) patient status (death, hospital readmission) at 12 months (only in England and Wales).

### Data collection

This trial is coordinated from the London School of Hygiene & Tropical Medicine (LSHTM) and will be conducted in hospitals worldwide. Data are collected at each site by local investigators and transmitted to the TCC. Only data outlined on the Entry, Outcome and Adverse event forms are collected for this trial.

Relevant data are recorded on the Entry form before randomisation to assess eligibility and the form completed if the patient is randomised. The Outcome form should be completed at death, discharge from the randomising hospital, or 28 days after randomisation, whichever occurs first. This data should be collected from the patient’s routine medical records as no special tests are required.

If the patient (or his/her PeR or PrR) withdraws a previously given informed consent or refuses to consent for continuation in the trial, or if the patient dies and no consent is available from either a PeR/PrR, his/her data will be handled as follows: data collected to the point of withdrawal of consent will be used as part of the intention-to-treat analysis; all relevant adverse events identified will be reported as required to all relevant authorities.

To allow for variation in available technology for data transfer, a variety of methods are used in this trial. Data are collected by the investigator on paper case report forms and transmitted to the TCC either as a paper form (by fax or email) or by entering the data directly into the trial database. The data are used in accordance with local law and ethics committee approval.

In England and Wales, patient identifiable information, including patient’s name, date of birth, NHS number and postcode, will be collected to allow trial staff based at LSHTM to follow up the patients’ progress at 12 months after randomisation. Follow-up will be done by linking this personal information to Hospital Episode Statistics through the Trusted Data Linkage Service of the NHS Information Centre for England and to Patient Episode Database for Wales through the Secure Anonymised Information Linkage Databank. Consent will be obtained before personal data are collected for the trial. The data will be treated in accordance with the Caldicott Principles and the Data Protection Act 1998. Access to the data will be restricted to authorised users and controlled and stored in accordance with the Act. All patient identifiable information will be stored at the TCC for a maximum of 10 years after the trial ends. These data are for follow-up purposes only and will not be held in the clinical trial database and will not be included in any analyses or publications.

### Monitoring

ICH-GCP section 5.18.3 states, in regard to monitoring, “The determination of the extent and nature of monitoring should be based on considerations such as the objective, purpose, design, complexity, blinding, size and endpoints of the trial. In general there is a need for on-site monitoring, before, during, and after the trial; however in exceptional circumstances the sponsor may determine that central monitoring in conjunction with procedures such as investigators training and meetings, and extensive written guidance can assure appropriate conduct of the trial in accordance with GCP. Statistically controlled sampling may be an acceptable method for selecting the data to be verified”.

This trial is a pragmatic, randomised, placebo-controlled trial. The intervention (TXA) has marketing authorisation in many countries and has been in clinical use for decades. The trial collects data on adverse events which may be associated with this product and the condition under investigation, and these will be reviewed routinely by the independent DMC. The trial involves getting consent, giving the trial drug in the usual way and collecting brief information from the hospital notes. There are no extra tests or procedures. Apart from the trial drug, all other treatment is as per usual practice. For these reasons, we believe that the risk of harm or injury (whether physical, psychological, social or economic) to trial participants is low. We use central monitoring along with investigators’ training and meetings, and extensive written guidance to make sure the trial is carried out properly. Statistically controlled sampling is used to select data to be verified. We plan to carry out on-site monitoring for about 10% of the trial data.

Consent forms from trial sites are monitored at the TCC but only where we have the written consent of the patients to do so.

Investigators/institutions are required to provide direct access to source data/documents for trial-related monitoring, audits, ethics committee review and regulatory inspection. All trial-related and source documents must be kept for 5 years after the end of the trial.

### End of trial for participants

Follow-up of the trial participants ends either at death, discharge, or 28 days post-randomisation, whichever occurs first. Adverse event reporting continues up to day 28.

In England and Wales, we will assess outcomes for participants at 12 months after the date of randomisation using routine data on mortality and hospital readmissions. We will include patient identifiers in the trial dataset to allow follow-up for deaths and for record linkage with mortality and hospital episode data.

The trial may be terminated early by the Trial Steering Committee (TSC). The DMC may give advice/recommendation for the early termination of the trial but the TSC is responsible for the final decision.

### Analysis

The main analyses will compare all those allocated TXA with those allocated placebo on an intention-to-treat basis. Results will be presented as effect estimates with a measure of precision (95% CI). Subgroup analyses for the primary outcome will be based on time to treatment, source of bleeding (upper versus lower), suspected variceal bleeding and severity of bleeding. Interaction tests will be used to explore whether the effect of treatment (if any) differs across these subgroups. A detailed statistical analysis plan setting out full details of the proposed analyses will be finalised before the trial database is locked for final analysis.

## Discussion

Acute GI bleeding is an emergency and an important cause of mortality and morbidity worldwide. Although clinical management has improved in recent years, mortality remains high. New effective treatments for patients with this condition are needed.

Early administration of TXA has been shown to reduce mortality in bleeding trauma patients; however, it is uncertain whether these results should be extrapolated from trauma to GI bleeding [17]. Patients with acute GI bleeding are usually older and have a high baseline risk of thromboembolic events. It is possible that harms from the use of TXA might be greater in patients with this condition compared to trauma patients.

A recent update of a systematic review identified nine randomised comparisons from eight clinical trials of the use of TXA in upper GI bleeding, and none in lower GI bleeding [20]. The pooled results showed a reduction in the risk of death in patients receiving TXA. However, the poor methodological quality of some of the studies and the inadequate sample size of trials, even when combined in the meta-analysis, suggests the possibility of an unreliable result. Moreover, only three trials reported data on adverse events. Specifically, the risk of thromboembolic events was about 1% overall and may be higher in TXA-treated patients [21].

The HALT-IT trial offers the opportunity to generate high-quality evidence on the effectiveness and safety of TXA in patients with GI bleeding. Currently, the use of TXA for GI bleeding is not supported by evidence and there are uncertainties about its safety and effectiveness. Only evidence coming from a high-quality, adequately powered, clinical trial will solve the uncertainty and improve clinical practice.

### Sponsorship and trial management

The HALT-IT trial is sponsored by the LSHTM and its responsibilities coordinated by the TCC. The TCC may delegate responsibilities to third parties which will be outlined in relevant agreements. The responsibilities of the TCC are overseen by the Trial Management Group (TMG).

### Indemnity

LSHTM accepts responsibility attached to its sponsorship of the trial and, as such, would be responsible for claims for any non-negligent harm suffered by anyone as a result of participating in this trial. The indemnity is renewed on an annual basis and LSHTM assures that it will continue renewal of the indemnity for the duration of this trial.

### Protocol development

The protocol Committee consists of the following investigators (Table 1) who are responsible for the development of and agreeing to the final protocol. Subsequent changes to the final protocol will require the agreement of the TSC.

**Table 1:** **Members of the protocol committee**

**Timothy Coats**, Emergency Medicine, University of Leicester, Leicester, UK	**Daniela Manno**, Clinical Lecturer, Clinical Trials Unit, London School of Hygiene & Tropical Medicine, London, UK
**Phil Edwards**, Senior Lecturer, Clinical Trials Unit, London School of Hygiene & Tropical Medicine, London, UK	**Ian Roberts**, Chief Investigator, Clinical Trials Unit, London School of Hygiene & Tropical Medicine, London, UK
**Ian Gilmore**, Consultant Gastroenterologist, University of Liverpool, Liverpool, UK	**Haleema Shakur**, Senior Lecturer, Clinical Trials Unit, London School of Hygiene & Tropical Medicine, London, UK
**Vipul Jairath**, National Institute for Health Research Clinical Lecturer, University of Oxford, Oxford, UK	**Simon Stanworth**, Consultant Haematologist, John Radcliffe Hospital, Oxford, UK
**Katharine Ker**, Lecturer, Clinical Trials Unit, London School of Hygiene & Tropical Medicine, London, UK	**Andrew Veitch**, Consultant Gastroenterologist, New Cross Hospital, Wolverhampton, UK

### Independent Data Monitoring Committee

An independent DMC has been appointed for this trial to oversee the safety monitoring (Table 2). The DMC will review on a regular basis accumulating data from the ongoing trial and advise the TSC regarding the continuing safety of current participants and those yet to be recruited, as well as reviewing the validity and scientific merit of the trial.

**Table 2:** **Composition of the independent Data Monitoring Committee (DMC)**

Name	Affiliation	Expertise
Professor Alan Barkun	McGill University, Canada	Clinical expert
Mr Tony Brady	Sealed Envelope Ltd, UK	Independent Statistician
Dr Philip Devereaux	McMaster University, Canada	Trials expert
Professor Richard Gray	Oxford University, UK	Statistician
Professor David Suresh	Christian Medical College Vellore, India	Clinical expert

The DMC composition, name, title and address of the chairman and of each member, is given in the DMC Charter which is in line with that proposed by the DAMOCLES Study Group [39]. Membership includes expertise in the relevant field of study, statistics and research study design.

The DMC Charter includes, but is not limited to, defining: 1) the schedule and format of the DMC meetings; 2) the format for presentation of data; 3) the method and timing of providing interim reports; and 4) stopping rules.

### Standard operating procedures

The DMC is independent from the sponsor, ethics committees, regulatory agencies, investigators, steering committee membership, clinical care of the trial patients, and any other capacity related to trial operations. The DMC has the responsibility for deciding whether, while randomisation is in progress, the unblinded results (or the unblinded results for a particular subgroup) should be revealed to the TSC. The DMC Charter states that they will do this if, and only if, two conditions are satisfied: (1) the results provide proof beyond reasonable doubt that treatment is on balance either definitely harmful or definitely favourable for all, or for a particular category of, participants in terms of the major outcome; (2) the results, if revealed, would be expected to substantially change the prescribing patterns of clinicians who are already familiar with any other trial results that exist. Exact criteria for ‘proof beyond reasonable doubt’ are not, and cannot be, specified by a purely mathematical stopping rule, but they are strongly influenced by such rules. DMC Charter is in agreement with the Peto-Haybittle [40, 41] stopping rule whereby an interim analysis of major endpoint would generally need to involve a difference between treatment and control of at least three standard errors to justify premature disclosure. An interim subgroup analysis would, of course, have to be even more extreme to justify disclosure. This rule has the advantage that the exact number and timing of interim analyses need not be pre-specified. In summary, the stopping rules require extreme differences to justify premature disclosure and involve an appropriate combination of mathematical stopping rules and scientific judgment.

### Trial Steering Committee

The role of the TSC is to provide overall supervision of the trial. In particular, the TSC concentrates on the progress of the trial, adherence to the protocol, patient safety and consideration of new information. The TSC must be in agreement with the final protocol and, throughout the trial, takes responsibility for: 1) major decisions such as a need to change the protocol for any reason; 2) monitoring and supervising the progress of the trial; 3) reviewing relevant information from other sources; 4) considering recommendations from the DMC; and 5) informing and advising the TMG on all aspects of the trial.

The TSC includes an experienced gastroenterologist, clinical trialists, chief investigator, clinical representative from a low- and middle-income country, and a patient representative (Table 3). Face-to-face meetings or teleconferences are held at regular intervals determined by need, but no less than once a year. A TSC Charter, which details how it conducts its business, has been agreed at the first meeting.

**Table 3:** **Composition of the Trial Steering Committee (TSC)**

Name	Affiliation	Expertise
Professor Christopher Hawkey	University of Nottingham, UK	Gastroenterologist and Chair of Trial Steering Committee
Dr Adefemi Afolabi	University of Ibadan, Nigeria	General Surgeon
Ms Barbara Farrell	University of Oxford, UK	Trials expert
Mr Ken Halligan	UK	Patient representative
Professor David Henry	Institute for Clinical Evaluative Sciences, Canada	Trials expert
Dr Chris Metcalfe	University of Bristol, UK	Statistician
Professor Ian Roberts	London School of Hygiene & Tropical Medicine, UK	Trials expert

When outcome data are available for 1,000 trial participants, the TSC will review the rate of recruitment into the trial and the overall event rates. The TSC will consider the extent to which the rate of recruitment and the event rates correspond to those anticipated before the trial and will take whatever action is needed in light of this information.

### Collaborators’ responsibilities

Coordination within each participating hospital is through a local Principal Investigator whose responsibility is detailed in an agreement in advance of starting the trial and includes: 1) ensure all necessary approvals are in place prior to starting the trial; 2) delegate trial related responsibilities only to suitably trained and qualified personnel; 3) train relevant medical and nursing staff who see gastroenterology patients and ensure that they remain aware of the state of the current knowledge, the trial and its procedures (there are wall charts, pocket summaries and PowerPoint presentations to assist with this); 4) agree to comply with the final trial protocol and any relevant amendments; 5) ensure that all patients with GI bleeding are considered promptly for the trial; 6) ensure consent is obtained in line with local approved procedures; 7) ensure that the patient entry and outcome data are completed and transmitted to the TCC in a timely manner; 8) ensure the Investigator’s Study File is up-to-date and complete; 9) ensure all adverse events are reported promptly to the TCC; 10) accountability for trial treatments at their site; 11) ensure the trial is conducted in accordance with ICH-GCP and fulfils all national and local regulatory requirements; 12) allow access to source data for monitoring, audit and inspection; and 13) be responsible for archiving all original trial documents including data forms for 5 years after the end of the trial.

### Trial management group and trial coordinating centre responsibilities

The TMG consists of the protocol Committee members plus a trial manager, data manager and trial administrator.

The TCC acts on behalf of the Sponsor and is responsible to the TMG to ensure that all of the Sponsor’s responsibilities are carried out. The responsibilities include (but are not limited to): 1) report to the TSC; 2) maintain the Trial Master File; 3) identify trial sites; 4) confirm all approvals are in place before release of the trial treatment and the start of the trial at a site; 5) provide training about the trial; 6) provide study materials; 7) data management centre; 8) 24-hour advice and unblinding service; 9) give collaborators regular information about the progress of the study; 10) respond to any questions (for example, from collaborators) about the trial; 11) ensure data security and quality and observe data protection laws; 12) safety reporting; 13) ensure trial is conducted in accordance with the ICH-GCP; 14) statistical analysis; and 15) publication of trial results.

### Contacting the Trial Coordinating Centre in an emergency

For urgent enquiries, adverse event reporting and unblinding queries investigators can contact the 24-hour telephone service provided by the TCC. A central telephone number is given in the Investigator’s Study File and wall posters.

### Publication and dissemination of results

The trial protocol and results will be published in peer-reviewed journals. All publications will follow the Consolidated Standards or Reporting Trials statement [42]. Links to the publication will be provided in all applicable trial registers. Dissemination of results to patients will take place via the media, trial website (http://haltit.Lshtm.ac.uk) and relevant patient organisations. Collaborating investigators will play a vital role in disseminating the results to colleagues and patients.

The success of the trial depends entirely upon the collaboration of nurses and doctors in the participating hospitals and those who hold key responsibility for the trial. Hence, the credit for the study will be assigned to the key collaborator(s) from a participating site as it is crucial that those taking credit for the work have actually carried it out. The results of the trial will be reported first to trial collaborators.

### Financial support

The HALT-IT trial is funded by the National Institute for Health Research Health Technology Assessment programme. Funding for this trial covers trial materials, meetings and central organisational costs. The design and management of the study are entirely independent of the manufacturers of TXA, which is not a new product. Large trials of such drugs, involving many hospitals, are important for future patients, but are practicable only if those collaborating in them do so without payment (except for recompense of any minor local costs that may arise). Agreement for repayment of local costs will be made in advance.

## Trial status

The study has been actively recruiting since July 2013. End of recruitment is planned for 31 May 2017 with end of follow-up due 30 June 2017 (except England and Wales, where 1-year follow-up will be complete on 31 May 2018). Further information is available at http://haltit.Lshtm.ac.uk/.

## Electronic supplementary material

Additional file 1: Form 1: Entry form, pages 1 and 2. (PDF 479 KB)

Additional file 2: Form 2: Outcome form, pages 1 and 2. (PDF 661 KB)

Additional file 3: Form 3: Brief information leaflet for patients and relatives, page 1. (PDF 268 KB)

Additional file 4: Form 4: Information sheet for the patient and representative, pages 1–4. (PDF 293 KB)

Additional file 5: Form 5: Consent form for the patient and representative. (PDF 505 KB)

Additional file 6: Form 6: List of ethical bodies that approved the study (updated at 31 July 2014). (DOCX 78 KB)

## Abbreviations

DMC: Data Monitoring Committee
GI: gastrointestinal
GMP: Good Manufacturing Practice
ICH-GCP: International Conference on Harmonisation of Good Clinical Practice
LSHTM: London School of Hygiene & Tropical Medicine
NHS: National Health Service
PeR: personal representative
PrR: professional representative
RR: relative risk
SAE: serious adverse event/experience
TCC: Trial Coordinating Centre
TMG: Trial Management Group
TSC: Trial Steering Committee
TXA: tranexamic acid.
