# Supplementary material for: HALT-IT - tranexamic acid for the treatment of gastrointestinal bleeding: study protocol for a randomised controlled trial
Source: Trials. 2014 Nov 19;15:450. doi: 10.1186/1745-6215-15-450 (PMC4253634; doi:10.1186/1745-6215-15-450)
Supplement: Supplementary file 1 — Additional file 1: Form 1: Entry form, pages 1 and 2. (PDF 479 KB) [file 13063_2014_2322_MOESM1_ESM.pdf]

ENTRY

PLEASE COMPLETE 1–19 BEFORE RANDOMISING THE PATIENT

ABOUT THE HOSPITAL

**1.** Country	
**2.** Hospital code *(in your Study File)*	

ABOUT THE PATIENT *(please ensure all information below is contained in the medical records)*

**3.** Patient's initials	*first*	*last*				
**4.** Sex *(circle)*	**MALE**	**FEMALE**				
**5.** Age						
**6.** Time since onset of GI bleed symptoms	*hours*	*In relation to THIS acute episode only*				
**7.** Suspected location of GI bleed *(circle one)*	**UPPER**	**LOWER**				
**8.** Haematemesis **or** coffee-ground vomitus *(circle)*	**YES**	**NO**	*Also circle YES if presence of blood in nasogastric aspirate*			
**9.** Melaena **or** fresh blood per rectum *(circle)*	**YES**	**NO**	*Also circle YES if occult or gross blood present on rectal examination*			
**10.** Suspected variceal bleed? *(circle)*	**YES**	**NO**				
**11.** Systolic blood pressure	*mmHg*	*Most recent measurement prior to randomisation*				
**12.** Heart rate	*beats per minute*	*Most recent measurement prior to randomisation*				
**13.** Signs of shock present? *(circle)*	**YES**	**NO**	*Shock assessment based on clinical signs (eg low BP, tachycardia, falling urine output) that requires intervention (eg intravenous fluids)*			
**14.** Suspected current active bleeding? *(circle)*	**YES**	**NO**	*Clinical judgement after considering history, signs and symptoms*			
**15.** Major co-morbidities? *(circle all that apply)*	CARDIOVASCULAR	RESPIRATORY	LIVER	RENAL	MALIGNANCY	OTHER MAJOR CO-MORBIDITY
**16.** On anti-coagulant therapy? *(circle)*	**YES**	**NO**	**UNKNOWN**			
**17.** Emergency admission? *(circle)*	**YES**	**NO**	*If patient already hospitalised, circle 'No'*			

RANDOMISATION INFORMATION

(fully eligible if adult, significant upper or lower GI bleed, AND uncertainty about the use of an antifibrInolytic in that particular patient)

**18.** Eligible? *(circle)*	**YES**		**NO** *do not randomise, record on screening log*	
**19.** Consent for entry obtained from *(circle)*	**WAIVER**	**RELATIVE**	**OTHER REPRESENTATIVE**	**PATIENT**
**20.** Treatment pack number *Take lowest available number treatment pack*	**BOX**		**PACK**	
**21.** Date of randomisation	*day*	*month*	*year*	
**22.** Time of randomisation *(24-hour clock)*	*hours*	*minutes*		
**23.** a) Name of person randomising patient	*first name*		*last name*	
b) Signature				

PLEASE SEND THESE DATA TO THE COORDINATING CENTRE **IMMEDIATELY** AFTER RANDOMISATION – **SEE GUIDANCE OVERLEAF**

DATA FORMS GUIDANCE

AFTER COMPLETING THIS PAPER FORM, YOU CAN:

- Enter these data directly into the trial database. For username and password, please contact **haltit.data@Lshtm.ac.uk**
- Send as a secure scanned document by email to **haltit.data@Lshtm.ac.uk** or upload a scanned copy at **http://ctu-files.Lshtm.ac.uk**.
- Fax to **020 7299 4663**
- Store original form in the Investigator’s Study File Section 15.
- **PLEASE GIVE A COPY OF THIS COMPLETED FORM TO THE PERSON RESPONSIBLE FOR COMPLETING THE OUTCOME FORM AT YOUR HOSPITAL**

NOTES:

FOR ADVERSE EVENTS, UNBLINDING AND OTHER URGENT ENQUIRIES PLEASE TELEPHONE +44(0)7768 707500

PLEASE NOTE: IF YOUR QUERY IS NOT URGENT PLEASE USE THE NORMAL CONTACT DETAILS IN THE INVESTIGATOR’S STUDY FILE AND WALL POSTERS
