# Supplementary material for: HALT-IT - tranexamic acid for the treatment of gastrointestinal bleeding: study protocol for a randomised controlled trial
Source: Trials. 2014 Nov 19;15:450. doi: 10.1186/1745-6215-15-450 (PMC4253634; doi:10.1186/1745-6215-15-450)
Supplement: Supplementary file 2 — Additional file 2: Form 2: Outcome form, pages 1 and 2. (PDF 661 KB) [file 13063_2014_2322_MOESM2_ESM.pdf]

Haltsit

OUTCOME

Complete at discharge from the randomising hospital, death in hospital or 28 days after randomisation, whichever occurs first

Attach treatment pack sticker or write box/pack number: ____ / ____

1. HOSPITAL

**a)** Country	
**b)** Hospital code	

2. PATIENT DETAILS

**a)** Initials	*first*	*last*
**b)** Age at entry		
**c)** Written consent obtained from patient or representative?	YES	NO
**d)** If no written consent, give reason		

3. PATIENT STATUS

3.1 Death in hospital *(if yes complete below – if no complete 3.2)*

**a)** Date of death	*dd*	*mm*	*yyyy*
**b)** Time of death *(24-hr clock)*	*hours*	*minutes*	

c) Main cause of death *(tick one option only)*

- ☐ Haemorrhage
- ☐ Malignancy
- ☐ Myocardial infarction
- ☐ Pneumonia
- ☐ Stroke
- ☐ Pulmonary embolism
- ☐ Other *(describe, 1 diagnosis only)* ____________________

3.2 Patient alive *(if yes complete **one** section below – if no complete 3.1)*

**a)** Discharged from hospital? *(Date)*	*dd*	*mm*	*yyyy*
**b)** Still in hospital at day 28? *(Date)*	*dd*	*mm*	*yyyy*

4. PROCEDURES *(circle one option on each line)*

**a)** Diagnostic endoscopic procedure	YES	NO
**b)** Therapeutic endoscopic procedure	YES	NO
**c)** Diagnostic radiological procedure	YES	NO
**d)** Therapeutic radiological procedure	YES	NO
**e)** Surgical intervention	YES	NO

5. PRIMARY CAUSE OF BLEED *(tick one option only)*

Upper GI Bleed	Lower GI Bleed
☐ Erosion or peptic ulcer	☐ Diverticular disease
☐ Varices	☐ Colitis
☐ Vascular lesion	☐ Vascular lesion
☐ Malignancy	☐ Malignancy
☐ Other/unknown	☐ Infection
	☐ Other/unknown

6. TRIAL TREATMENT *(only circle YES if complete dose given)*

**a)** Loading dose given	YES	NO
**b)** Maintenance dose given	YES	NO

7. OTHER TREATMENTS *(circle one option on each line)*

**a)** Helicobacter pylori eradication	YES	NO
**b)** H2 receptor antagonists	YES	NO
**c)** Proton pump inhibitors	YES	NO
**d)** Vasopressin / analogue	YES	NO
**e)** Antibiotics for variceal bleeding	YES	NO
**f)** Antifibrinolytics	YES	NO

8. BLOOD PRODUCTS TRANSFUSION *(if none enter 0)*

**a)** Were blood products transfused?	YES	NO
**b)** Units whole blood/red cells *(part unit = 1 unit)*	*units*	
**c)** Frozen plasma *(part unit = 1 unit)*	*units*	
**d)** Platelets *(part unit = 1 unit)*	*units*	

9. MANAGEMENT *(if none enter 0)*

**a)** Days in Intensive Care Unit (ICU)	*days*
**b)** Days in High Dependency Unit (HDU)	*days*

10. COMPLICATIONS *(circle one option on each line)*

**a)** Re-bleeding	YES	NO
**b)** Deep vein thrombosis	YES	NO
**c)** Pulmonary embolism	YES	NO
**d)** Stroke	YES	NO
**e)** Myocardial infarction	YES	NO
**f)** Other significant cardiac event	YES	NO
**g)** Sepsis	YES	NO
**h)** Pneumonia	YES	NO
**i)** Respiratory failure	YES	NO
**j)** Liver failure	YES	NO
**k)** Renal failure	YES	NO
**l)** Seizures	YES	NO

Any complications not listed above – please report as per protocol using an Adverse Event Reporting form.

11. PATIENT'S SELF CARE CAPACITY

(circle one option on each line)

	INDEPENDENT?	
**a) Bathing** *(sponge bath, tub bath, or shower) – Receives either no assistance or assistance in bathing only one part of body*	YES	NO
**b) Dressing** *– Gets clothed and dressed without assistance except for tying shoes*	YES	NO
**c) Toileting** *– Goes to toilet room, uses toilet, arranges clothes, and returns without assistance (may use cane or walker for support and bedpan/urinal at night)*	YES	NO
**d) Transferring** *– Moves in and out of bed and chair without assistance (may use cane or walker)*	YES	NO
**e) Continence** *– Controls bowel and bladder completely by self (without occasional 'accidents')*	YES	NO
**f) Feeding** *– Feeds self without assistance (except for help with cutting meat or buttering bread)*	YES	NO

UK ONLY – PATIENT IDENTIFIERS

**a)** Name	*first name*	*family name*	
**b)** Date of birth	*dd*	*mm*	*yyyy*
**c)** Post code			
**d)** NHS number			

12. PERSON COMPLETING FORM *(PI is responsible for data submitted)*

**a)** Name	*first name*	*last name*	
**b)** Position			
**c)** Signature			
**d)** Date	*dd*	*mm*	*yyyy*

SEE GUIDANCE NOTES ON REVERSE

DETAILED GUIDANCE ABOUT COMPLETING THIS FORM CAN BE FOUND IN YOUR INVESTIGATORS STUDY FILE

AFTER COMPLETING THIS PAPER FORM, YOU CAN:

- Enter these data directly into the trial database. For username and password, please contact **haltit.data@Lshtm.ac.uk**
- Send as a secure scanned document by email to **haltit.data@Lshtm.ac.uk** or upload a scanned copy at **http://ctu-files.Lshtm.ac.uk**.
- Fax to **+44 20 7299 4663**

STORE THIS ORIGINAL FORM IN YOUR SITE FILE
