# Supplementary material for: HALT-IT - tranexamic acid for the treatment of gastrointestinal bleeding: study protocol for a randomised controlled trial
Source: Trials. 2014 Nov 19;15:450. doi: 10.1186/1745-6215-15-450 (PMC4253634; doi:10.1186/1745-6215-15-450)
Supplement: Supplementary file 3 — Additional file 3: Form 3: Brief information leaflet for patients and relatives, page 1. (PDF 268 KB) [file 13063_2014_2322_MOESM3_ESM.pdf]

THE HALT-IT TRIAL

BRIEF INFORMATION ABOUT THE RESEARCH

Haemorrhage alleviation with tranexamic acid - Intestinal system

Tranexamic acid for the treatment of gastrointestinal haemorrhage:
an international randomised, double blind placebo controlled trial

You (the patient) have bleeding from the gut that needs to be stopped quickly. All the usual treatments for the bleeding that we provide at this hospital will be given. As well as this, we are inviting you to take part in a study. This study will see if a treatment called *tranexamic acid* reduces gut bleeding. We hope that this treatment will lead to a better recovery. We know that the treatment helps people with other types of bleeding but we don't know yet if it works in gut bleeding.

In this study, half the patients will get the study treatment (*tranexamic acid*) and half a dummy treatment (a placebo). If you take part in this study, you (the patient) will receive the study treatment or placebo straight away. It will be given to you through a drip over 24 hours. We will also need to collect some information about your (the patient's) medical condition and send it to a central office in London.

If you would like to know more about our study now, then we will tell you. But otherwise we will tell you more about it later. Are you willing for us to go ahead with the study treatment?

Yes, I am willing for you to go ahead.

______________________________	________________	_________________________________
Name of Patient<br>or representative	Date	Signature (thumbprint or<br>other mark if unable to sign)

[This information can be presented verbally and does not need to be used verbatim. It can be adapted to each situation and is provided to be used only as a guide. A signed form is not mandatory and if signed, must not be viewed as a valid Informed Consent.]
