# Supplementary material for: HALT-IT - tranexamic acid for the treatment of gastrointestinal bleeding: study protocol for a randomised controlled trial
Source: Trials. 2014 Nov 19;15:450. doi: 10.1186/1745-6215-15-450 (PMC4253634; doi:10.1186/1745-6215-15-450)
Supplement: Supplementary file 4 — Additional file 4: Form 4: Information sheet for the patient and representative, pages 1–4. (PDF 293 KB) [file 13063_2014_2322_MOESM4_ESM.pdf]

THE HALT-IT TRIAL
INFORMATION ABOUT THE RESEARCH

Title of Research: Tranexamic acid for the treatment of gastrointestinal haemorrhage: an international randomised, double blind placebo controlled trial

This hospital is taking part in a study to find better treatments for gut bleeding

One of the following applies to you:

1) You are a patient with bleeding from your gut. We are inviting you to take part in this research study.
2) You are a patient who had bleeding from your gut. When you were very unwell you were included in this research study. We are now asking you to consider taking part in the rest of the study.
3) You are a representative of a patient who has bleeding from the gut. We are asking you to decide on the patient's behalf whether s/he can take part in this research study. You may wish to consider if you think that the patient would have agreed to take part if s/he had been well enough to decide.

What is this leaflet for?

Before you decide to take part in this study we would like you to know why it is being done and what it will involve.

One of our team will go through this leaflet with you and answer any questions you have. We think that this will take about 20 minutes.

You can talk to others about the study if you wish.

What is the study for?

We are looking to see if there is a better treatment for gut bleeding. We hope that the study treatment (a drug called tranexamic acid), which helps blood clotting, will reduce gut bleeding. But it is also possible that it could cause clots where they are not needed.

Tranexamic acid is not a new drug. It is already used to help people who are bleeding after an operation or an accident. We hope that this treatment will do more good than harm in patients who are bleeding from the gut but we don't yet know this for sure.

Why have I been asked to take part?

You (the patient) have bleeding from your gut that needs to be treated quickly. Your doctor thinks that you can join the study, but it is up to you to decide whether to take part or not.

If you do take part, you will be one of about 8,000 people with gut bleeding in this study from all over the world.

A patient cannot be in this study if:

- the doctor thinks there is a particular reason why tranexamic acid definitely **should not** be given
- the doctor thinks there is a particular reason why tranexamic acid definitely **should** be given
- he/she is not an adult

You (the patient) have been invited to take part in this study because none of the above conditions apply to you.

Do I have to take part?

No. It is up to you to decide to take part or not. If you don't want to take part, your doctor will still care for you and give you all the other treatments you need.

How does the study work?

We don't know if giving tranexamic acid as well as all the usual treatments for gut bleeding will be better or not. The best way to find out is to see how people who are given it do, compared to people who are not. To do this, the people taking part in the study will be put into one of two groups. One group will get the study treatment (tranexamic acid). The other group will get a dummy treatment (a placebo). Which group a person is put into is decided randomly and each person has an equal chance of being put into either group. The study treatment and the dummy treatment look the same, so you and your doctor will not know which group you are in.

What will happen to me if I take part?

You will get all the usual treatments to help your bleeding. You will also be given one of the study treatments (tranexamic acid or placebo). You will start this treatment straight away. It will be given to you through a drip over 24 hours. The study treatment is free. You will not need any extra tests or to spend longer in hospital because of the study.

We will give you this leaflet to keep and ask you to sign a consent form.

We would also like to send a letter to let your GP know that you are taking part in the study.

What will happen afterwards?

We will want to know about your health after leaving hospital. Before you go home you will be given a card to take with you. If you see a doctor or nurse for an illness within a month of coming into hospital, you should show them the card.

Will I be hurt by taking part?

Other studies suggest that the study treatment (tranexamic acid) doesn't cause unwanted blood clots and there are no bad side effects with short term use, but we do not know if this will be the same for people with your condition. Your doctor will watch you and give you the best available care if there are any problems. They will also tell the people running the study.

Will I gain from taking part?

We do not know if this study will help you. But it will help doctors treat people who have gut bleeding in the future.

Can I change my mind about taking part?

Yes. If you change your mind about taking part, you just need to tell your doctor that you don't want to be in the study any more. You can do this at any time. Your doctor will still care for you and give you all the other treatments you need. We hope that you will still let us use the information about how you got on, but if you do not want us to use it please tell your doctor.

What information will be collected about me?

Details about your bleeding, the medicines you get and how you get on will be written down. We will also look at how you are in one year by looking at NHS computer records. To do this we will need to write down your name, date of birth, home postcode and NHS number.

Staff at the main office in London may also want to collect some forms that have your name on, including a copy of your signed consent form. This will help them to ensure that the study is being carried out correctly.

Will my information be kept private?

All information collected about you will be kept private. People allowed to look at the information will be the doctors running the study, the staff at the main office in London and authorities who check that the study is being carried out properly. To check how you are doing in one year, we will need to send your personal details to the NHS centres that keep track of people who go to hospital in England and Wales.

Your doctor will send some details about you to the study team in London who will store it securely. Your personal details will be kept in a different safe place to the other study information and will be destroyed within five years of the end of the study.

The study results will be published in a medical journal so that other doctors can learn from them. Your personal information will not be included in the study report and there is no way that you can be identified from it.

The study data will be made available to researchers worldwide so that it can be used to improve medical knowledge and patient care. Your personal information will not be included and there is no way that you can be identified.

Who is in charge of this study?

The study is run by a team at the London School of Hygiene & Tropical Medicine at the University of London.

Who is paying for this study?

The study is paid for by the NHS. Your doctor is not being paid for including patients in this study.

Who has checked this study?

To look after your interests, this study has been carefully checked by an independent group of people called a Research Ethics Committee. They agreed that it is okay for us to ask people to take part.

Will I be able to find out what the study results are?

The study should end in the year 2018. If you would like to have a summary of the results of this study when it has ended, please let the doctor treating you know.

You can also visit the study’s website to see the progress of the study (haltit.Lshtm.ac.uk).

Who can I talk to if I have any other questions or concerns?

You can talk to your doctors and nurses about the study. They will do their best to answer your questions. You can also speak to Dr [insert name] who is in charge of this study at your hospital. You can write to [him/her] at [address] or phone on [number].

If you remain unhappy you can make a formal complaint through the NHS complaints procedure. Your doctor can give you details on how to do this.

What else do I need to know?

If something does go wrong and you are harmed during the study, and this is due to someone's negligence, then you may have grounds to seek compensation. The London School of Hygiene & Tropical Medicine, who are organising the study, would be responsible for claims for any non-negligent harm suffered as a result of taking part in this study. The NHS will be liable for clinical negligence and other negligent harm as a result of the clinical procedure being undertaken. You may have to pay your legal costs.

You are encouraged to ask any questions you wish, before, during or after the study. If you have any questions about the study, please speak to your study nurse or doctor, who will be able to provide you with up to date information about the drug(s)/procedure(s) involved. If you wish to read the research on which this study is based, please ask your study nurse or doctor.
