# Supplementary material for: HALT-IT - tranexamic acid for the treatment of gastrointestinal bleeding: study protocol for a randomised controlled trial
Source: Trials. 2014 Nov 19;15:450. doi: 10.1186/1745-6215-15-450 (PMC4253634; doi:10.1186/1745-6215-15-450)
Supplement: Supplementary file 5 — Additional file 5: Form 5: Consent form for the patient and representative. (PDF 505 KB) [file 13063_2014_2322_MOESM5_ESM.pdf]

CONSENT FORM FOR PATIENT AND REPRESENTATIVE

THE HALT-IT TRIAL

Title of Research: Tranexamic acid for the treatment of gastrointestinal haemorrhage: an international randomised, double blind placebo controlled trial

Hospital code		Local Principal Investigator							
Patient hospital ID number		Randomisation number							
			BOX				PACK		
Name of patient		If representative, relationship to patient							

Please INITIAL boxes

1. I have read and understood the information sheet (version number 1.2, date 22/03/2013) and have had a chance to ask questions. ☐
2. I understand that it is my choice to take part in this study. I am free to pull out at any time, without giving a reason and without my (the patient's) treatment or rights being affected. ☐
3. I understand that parts of my (the patient's) medical notes may be looked at by people involved in the study. I allow them to see my notes. ☐
4. I allow a copy of this form to be made available to the study staff in London for monitoring. ☐
5. I allow my (the patient's) name, date of birth, post code and NHS number to be sent to the study staff in London so they can find out how I am in one year's time. ☐
6. I allow my GP to be told that I (the patient) am taking part in this study. ☐
7. I give permission for the data collected about me in this trial (with my personal information removed) to be used by researchers worldwide. ☐
8. I agree to me (the patient) taking part in the above study, the HALT-IT trial. ☐

_______________________________ _______________ _______________________________
Name of patient/representative Date Signature (thumbprint or other mark if unable to sign)

_______________________________ _______________ _______________________________
Name of person taking consent Date Signature

_______________________________ _______________ _______________________________
Name of Principal Investigator Date Signature

The patient/representative is unable to sign. As a witness, I confirm that all the information about the trial was given and the patient/representative consented to taking part.

_______________________________ _______________ _______________________________
Name of witness Date Signature

Original to be filed in the Investigator's Study File, 1 copy for patient, 1 copy to be kept with patient's hospital records
