# Supplementary material for: HALT-IT - tranexamic acid for the treatment of gastrointestinal bleeding: study protocol for a randomised controlled trial
Source: Trials. 2014 Nov 19;15:450. doi: 10.1186/1745-6215-15-450 (PMC4253634; doi:10.1186/1745-6215-15-450)
Supplement: Supplementary file 6 — Additional file 6: Form 6: List of ethical bodies that approved the study (updated at 31 July 2014). (DOCX 78 KB) [file 13063_2014_2322_MOESM6_ESM.docx]

National research ethics committee that approved the protocol (updated at 31^st^ Jul 2014)

| **Name** | **Country** | **Approval granted** | **Reference number** |
| --- | --- | --- | --- |
| NRES Committee East of England - Essex | United Kingdom | 15/03/2013 | 12/EE/0038 |

Local research ethics committee that approved the protocol (updated at 31^st^ Jul 2014)

| **Name** | **Country** | **Site** | **Approval granted** | **Reference number** |
| --- | --- | --- | --- | --- |
| Hospital Universitario San Vicente Fundacion | Colombia | Hospital Universitario San Vicente Fundacion | 23/07/2014 |  |
| Centre of Emergency Surgery and Traumatology | Georgia | Centre of Emergency Surgery and Traumatology | 12/12/2013 |  |
| High Technology Medical Center, University Clinic | Georgia | High Technology Medical Center, University Clinic | 15/12/2013 |  |
| National Hospital Abuja HREC | Nigeria | National Hospital Abuja | 16/07/2013 | NHA/EC/226/2013 |
| Aminu Kano Teaching Hospital Health Research Ethics Committee | Nigeria | Aminu Kano Teaching Hospital | 13/06/2013 | AKTH/MAC/SUB/12A/P-3/VI/1158 |
| Federal Capital Territory HREC | Nigeria | Maitama District Hospital | 25/03/2014 | FHREC/2014/01/09/25-03-14 |
| Institutional Health Research Ethics Committee (Jos) | Nigeria | Jos University Teaching Hospital | 05/08/2013 | JUTH/DCS/ADM/127/XXII/5740 |
| Lagos University Teaching Hospital Health Research Ethics Committee | Nigeria | Lagos University Teaching Hospital - Enugu | 23/10/2013 | ADM/DCST/HREC/1127 |
| Olabisi Onabanjo University Teaching Hospital -HREC | Nigeria | Olabisi Onabanjo University Teaching Hospital | 20/06/2013 | OOUTH/DA.326/890 |
| University of Ilorin Teaching Hospital (UITH) Ethical Research Committee | Nigeria | University of Ilorin Teaching Hospital | 02/10/2013 | NHREC/02/05/2010 |
| University of Nigeria Teaching Hospital Health Research Ethics Committee | Nigeria | University of Nigeria Teaching Hospital, Enugu | 15/10/2013 | UNTH/CSA/329/Vol.5 |
| Aintree University Hospital NHS Trust R&D | United Kingdom | Aintree University Hospital | 04/12/2013 |  |
| Barnsley Hospital NHS trust R&D | United Kingdom | Barnsley Hospital | 01/05/2013 |  |
| Barts Health NHS Trust R&D | United Kingdom | Whipps Cross University Hospital | 09/07/2013 |  |
| Barts Health NHS Trust R&D | United Kingdom | Royal London Hospital | 09/07/2013 |  |
| Barts Health NHS Trust R&D | United Kingdom | Newham University Hospital | 09/07/2013 |  |
| Basildon and Thurrock University Hospitals NHS Trust R&D | United Kingdom | Basildon University Hospital | 13/02/2014 |  |
| Blackpool, Fylde and Wyre Hospitals NHS Foundation Trust R&D | United Kingdom | Blackpool Victoria Hospital | 24/09/2013 |  |
| Brighton and Sussex University Hospitals NHS trust R&D | United Kingdom | Royal Sussex County Hospital | 17/06/2013 |  |
| Cambridge University Hospitals NHS Foundation Trust R&D | United Kingdom | Addenbrooke's Hospital | 12/12/2013 |  |
| Cardiff & Vale University Health Board | United Kingdom | University Hospital of Wales | 11/09/2013 |  |
| Central Manchester University Hospitals NHS Trust R&D | United Kingdom | Manchester Royal Infirmary | 13/11/2013 |  |
| Chelsea and Westminster Hospital NHS trust R&D | United Kingdom | Chelsea and Westminster Hospital | 08/05/2013 |  |
| Colchester Hospital University NHS Foundation Trust R&D | United Kingdom | Colchester General Hospital | 05/12/2013 |  |
| County Durham and Darlington NHS Foundation Trust R&D | United Kingdom | Darlington Memorial Hospital | 20/11/2013 |  |
| Dorset County Hospital NHS Foundation Trust R&D | United Kingdom | Dorset County Hospital | 15/07/2013 |  |
| Gateshead NHS Foundation Trust R&D | United Kingdom | Queen Elizabeth Hospital - Gateshead | 01/07/2013 |  |
| Hampshire Hospitals NHS Foundation Trust R&D | United Kingdom | Basingstoke and North Hampshire Hospital | 14/10/2013 |  |
| Hampshire Hospitals NHS Foundation Trust R&D | United Kingdom | Royal Hampshire County Hospital | 24/04/2014 |  |
| Heatherwood and Wexham Park Hospitals NHS Foundation trust R&D | United Kingdom | Wexham Park Hospital | 05/07/2013 |  |
| Hospitals Coventry & Warwickshire NHS Trust R&D | United Kingdom | University Hospital Coventry | 07/05/2014 |  |
| Hull and East Yorkshire Hospitals NHS Trust R&D | United Kingdom | Hull Royal Infirmary | 05/11/2013 |  |
| Ipswich Hospital R&D | United Kingdom | Ipswich Hospital | 26/09/2013 |  |
| King's College Hospital NHS Foundation Trust R&D | United Kingdom | King's College Hospital | 25/04/2014 |  |
| Leeds Teaching Hospitals NHS Trust R&D | United Kingdom | St James University Hospital - Leeds | 06/02/2014 |  |
| LSHTM Research Ethics Committee | United Kingdom | LSHTM | 19/12/2012 | 6328 |
| Newcastle Hospitals NHS Foundation Trust R&D | United Kingdom | Royal Victoria Infirmary | 13/09/2013 |  |
| NHS Ayrshire and Arran R&D | United Kingdom | University Hospital Ayr | 07/11/2013 |  |
| NHS Ayrshire and Arran R&D | United Kingdom | University Hospital Crosshouse | 11/11/2013 |  |
| NHS Grampian R&D | United Kingdom | Aberdeen Royal Infirmary | 06/05/2014 |  |
| NHS Greater Glasgow and Clyde R&D | United Kingdom | Glasgow Royal Infirmary | 27/08/2013 |  |
| NHS Lothian R&D | United Kingdom | Royal Infirmary of Edinburgh | 06/06/2013 |  |
| North Bristol NHS Trust R&D | United Kingdom | Southmead Hospital (former Frenchay Hospital) | 20/09/2013 |  |
| North Cumbria University Hospitals NHS Trust R&D | United Kingdom | Cumberland Infirmary Hospital | 20/05/2013 |  |
| North Cumbria University Hospitals NHS Trust R&D | United Kingdom | West Cumberland Hospital | 20/05/2013 |  |
| North Tees and Hartlepool NHS Foundation Trust R&D | United Kingdom | University Hospital of North Tees | 19/07/2013 |  |
| Northampton General Hospital NHS Trust R&D | United Kingdom | Northampton General Hospital | 12/11/2013 |  |
| Northern Devon Healthcare NHS Trust R&D | United Kingdom | North Devon District Hospital | 20/06/2013 |  |
| Nottingham University Hospitals NHS Trust R&D | United Kingdom | Queen's Medical Centre Campus - Nottingham | 06/06/2013 |  |
| Oxford University Hospitals NHS Trust R&D | United Kingdom | John Radcliffe Hospital | 02/07/2013 |  |
| Pennine Acute Hospitals NHS Trust R&D | United Kingdom | Royal Oldham Hospital | 24/01/2014 |  |
| Plymouth Hospitals NHS Trust R&D | United Kingdom | Derriford Hospital - Plymouth | 09/07/2014 |  |
| Poole Hospital NHS Foundation Trust R&D | United Kingdom | Poole Hospital | 13/06/2013 |  |
| Royal Berkshire NHS Foundation Trust R&D | United Kingdom | Royal Berkshire Hospital | 07/06/2013 |  |
| Royal Bournemouth and Christchurch Hospitals NHS Foundation Trust R&D | United Kingdom | Royal Bournemouth Hospital | 16/10/2013 |  |
| Royal Devon and Exeter NHS Foundation Trust R&D | United Kingdom | Royal Devon & Exeter Hospital | 14/06/2013 |  |
| Royal Liverpool and Broadgreen University Hospitals NHS Trust R&D | United Kingdom | Royal Liverpool University Hospital | 28/02/2014 |  |
| Royal Shrewsbury Hospitals NHS Trust R&D | United Kingdom | Royal Shrewsbury Hospital | 25/07/2013 |  |
| Royal United Hospital Bath NHS Trust R&D | United Kingdom | Royal United Hospital Bath | 11/11/2013 |  |
| Salford Royal NHS Foundation Trust R&D | United Kingdom | Salford Royal Hospital | 29/08/2013 |  |
| Salisbury NHS Foundation Trust R&D | United Kingdom | Salisbury District Hospital | 16/08/2013 |  |
| Sandwell and West Birmingham NHS Trust R&D | United Kingdom | Birmingham City Hospital | 17/12/2013 |  |
| Sandwell and West Birmingham NHS Trust R&D | United Kingdom | Sandwell General Hospital | 17/12/2013 |  |
| Sheffield Teaching Hospitals NHS Foundation Trust R&D | United Kingdom | Northern General Hospital | 14/04/2014 |  |
| South Devon Healthcare NHS Foundation Trust R&D | United Kingdom | Torbay Hospital - Devon | 30/06/2014 |  |
| South Tees Hospitals NHS Foundation Trust R&D | United Kingdom | James Cook University Hospital | 06/01/2014 |  |
| South Tyneside NHS Foundation Trust R&D | United Kingdom | South Tyneside District Hospital | 09/07/2013 |  |
| St George's Healthcare NHS Trust R&D | United Kingdom | St George's Hospital | 05/11/2013 |  |
| Taunton and Somerset NHS Foundation Trust R&D | United Kingdom | Musgrove Park Hospital | 20/01/2014 |  |
| The Royal Wolverhampton Hospitals NHS Trust R&D | United Kingdom | New Cross Hospital | 03/07/2013 |  |
| University College London Hospitals NHS Foundation Trust R&D | United Kingdom | University College London Hospital | 19/11/2013 |  |
| University Hospital of North Staffordshire NHS Trust R&D | United Kingdom | University Hospital of North Staffordshire | 10/10/2013 |  |
| University Hospital Southampton NHS Foundation Trust R&D | United Kingdom | Southampton General Hospital | 09/01/2014 |  |
| University Hospitals Birmingham NHS Foundation Trust R&D | United Kingdom | Queen Elizabeth Hospital - Birmingham | 27/06/2013 |  |
| University Hospitals of Leicester NHS Trust R&D | United Kingdom | Leicester Royal Infirmary | 27/08/2013 |  |
| University Hospitals of Morecambe Bay NHS Foundation Trust R&D | United Kingdom | Royal Lancaster Infirmary | 21/10/2013 |  |
| Western Sussex Hospitals NHS Foundation Trust R&D | United Kingdom | Worthing Hospital | 01/11/2013 |  |
| Whittington NHS Trust R&D | United Kingdom | Whittington Hospital | 21/11/2013 |  |
| Worcestershire Acute Hospitals NHS Trust R&D | United Kingdom | Alexandra Hospital - Redditch | 23/07/2014 |  |
| Yeovil District Hospital NHS Foundation Trust R&D | United Kingdom | Yeovil District Hospital | 10/07/2013 |  |
| York Foundation Trust R&D | United Kingdom | York Hospital | 02/10/2013 |  |
